# Context-sensitive autoassociative memories as expert systems in medical diagnosis

**DOI:** 10.1186/1472-6947-6-39

**Published:** 2006-11-22

**Authors:** Andrés Pomi, Fernando Olivera

**Affiliations:** 1 Sección Biofísica, Facultad de Ciencias, Universidad de la República, Iguá 4225, 11400 Montevideo, Uruguay; 2 Departamento de Biofísica, Facultad de Medicina, Universidad de la República, General Flores 2125,11800 Montevideo, Uruguay

## Abstract

**Background:**

The complexity of our contemporary medical practice has impelled the development of different decision-support aids based on artificial intelligence and neural networks. Distributed associative memories are neural network models that fit perfectly well to the vision of cognition emerging from current neurosciences.

**Methods:**

We present the context-dependent autoassociative memory model. The sets of diseases and symptoms are mapped onto a pair of basis of orthogonal vectors. A matrix memory stores the associations between the signs and symptoms, and their corresponding diseases. A minimal numerical example is presented to show how to instruct the memory and how the system works. In order to provide a quick appreciation of the validity of the model and its potential clinical relevance we implemented an application with real data. A memory was trained with published data of neonates with suspected late-onset sepsis in a neonatal intensive care unit (NICU). A set of personal clinical observations was used as a test set to evaluate the capacity of the model to discriminate between septic and non-septic neonates on the basis of clinical and laboratory findings.

**Results:**

We show here that matrix memory models with associations modulated by context can perform automatic medical diagnosis. The sequential availability of new information over time makes the system progress in a narrowing process that reduces the range of diagnostic possibilities. At each step the system provides a probabilistic map of the different possible diagnoses to that moment. The system can incorporate the clinical experience, building in that way a representative database of historical data that captures geo-demographical differences between patient populations. The trained model succeeds in diagnosing late-onset sepsis within the test set of infants in the NICU: sensitivity 100%; specificity 80%; percentage of true positives 91%; percentage of true negatives 100%; accuracy (true positives plus true negatives over the totality of patients) 93,3%; and Cohen's kappa index 0,84.

**Conclusion:**

Context-dependent associative memories can operate as medical expert systems. The model is presented in a simple and tutorial way to encourage straightforward implementations by medical groups. An application with real data, presented as a primary evaluation of the validity and potentiality of the model in medical diagnosis, shows that the model is a highly promising alternative in the development of accuracy diagnostic tools.

## Background

The extreme complexity of contemporary medical knowledge together with the intrinsic fallibility of human reasoning, have led to sustained efforts to develop clinical decision support systems, with the hope that bedside expert systems could overcome the limitations inherent to human cognition [1]. Despite the foundational hopes have not been fulfilled [2], the unaltered and increasing necessity for reliable automated diagnostic tools and the important benefit to society brought by any success in this area make every advance valuable.

To further the research on computer-aided diagnosis begun in the 1960s, models of neural networks [3] have been added to the pioneering work on artificial-intelligence systems. The advent of artificial neural networks with ability to identify multidimensional relationships in clinical data might improve the diagnostic power of the classical approaches. A great proportion of the neural network architectures applied to clinical diagnosis rests on multilayer feed-forward networks instructed with backpropagation, followed by self-organizing maps and ART models [4,5]. Although they perform with significant accuracy, this performance nevertheless remained insufficient to dispel the common fear that they are "black-boxes" whose functioning cannot be well understood and, consequently, whose recommendations cannot be trusted [6].

The associative memory models, an early class of neural models [7] that fit perfectly well with the vision of cognition emergent from today brain neuroimaging techniques [8,9], are inspired on the capacity of human cognition to build semantic nets [10]. Their known ability to support symbolic calculus [11] makes them a possible link between connectionist models and classical artificial-intelligence developments.

This work has three main objectives: a) to point out that associative memory models have the possibility to act as expert systems in medical diagnosis; b) to show in a simple and straightforward way how to instruct a minimal expert system with associative memories; and c) to encourage the implementation of this methodology at large scale by medical groups.

Therefore, in this paper we address – in a tutorial approach – the building of associative memory-based expert systems for the medical diagnosis domain. We favour a comprehensive way and the possibility of a straightforward implementation by medical groups over the mathematical details of the model.

## Methods

### Context-dependent autoassociative memories with overlapping contexts

Associative memories are neural network models developed to capture some of the known characteristics of human memories [12,13]. These memories associate arbitrary pairs of patterns of neuronal activity mapped onto real vectors. The set of associated pairs is stored superimposed and distributed throughout the coefficients of a matrix. These matrix memory models are content-addressable and fault-tolerant, and are well known to share with humans the ability of generalization and universalization [14].

In the attempt to overcome a serious problem of these classical models – their impossibility to evoke different associations depending on the context accompanying a same key stimulus- Mizraji [15] developed an extension of the model that performs adaptive associations. Context-dependent associations are based on a kind of second order sigma-pi neurons [16], and showed an interesting versatility when they were incorporated in modules employed to implement chains of goal-directed associations [17], disambiguation of complex stimuli [18], logical reasoning [19,20], and multiple criteria classification [21].

A context-dependent associative memory M acting as a basic expert system is a matrix

M=∑i=1kdi(di⊗∑j(i)sj)T     (1)
 MathType@MTEF@5@5@+=feaafiart1ev1aaatCvAUfKttLearuWrP9MDH5MBPbIqV92AaeXatLxBI9gBaebbnrfifHhDYfgasaacH8akY=wiFfYdH8Gipec8Eeeu0xXdbba9frFj0=OqFfea0dXdd9vqai=hGuQ8kuc9pgc9s8qqaq=dirpe0xb9q8qiLsFr0=vr0=vr0dc8meaabaqaciaacaGaaeqabaqabeGadaaakeaacqqGnbqtcqGH9aqpdaaeWbqaaiabbsgaKnaaBaaaleaacqqGPbqAaeqaaOGaeiikaGIaeeizaq2aaSbaaSqaaiabbMgaPbqabaGccqGHxkcXdaaeqbqaaiabbohaZnaaBaaaleaacqqGQbGAaeqaaOGaeiykaKYaaWbaaSqabeaacqqGubavaaaabaGaeeOAaOMaeiikaGIaeeyAaKMaeiykaKcabeqdcqGHris5aaWcbaGaeeyAaKMaeyypa0JaeGymaedabaGaee4AaSganiabggHiLdGccaWLjaGaaCzcamaabmaabaGaeGymaedacaGLOaGaayzkaaaaaa@4DD7@

where d_i _are column vectors mapping k different diseases (the set {d} is chosen orthonormal), and s_j(i) _are column vectors mapping signs or symptoms accompanying the i disease (also an orthonormal set). The sets of symptoms corresponding to each disease can overlap.

The Kronecker product (⊗) between two matrices A and B is another matrix defined by

A ⊗ B = a(i, j)·B     (2)

denoting that each scalar coefficient of matrix A, a(i, j), is multiplied by the entire matrix B. Hence, if A is nxm dimensional and B is kxl dimensional, the resultant matrix will have the dimension nkxml.

Note that if d are n-dimensional and s are k-dimensional vectors, the memory is a rectangular nxnm matrix. Also, the memory M can be viewed as resulting from the Kronecker product (⊗) *enlargement *of each element of a nxn square autoassociative matrix d_i _d_i_^T^ by a row column representing the sum of corresponding signs and symptoms:

M=∑i=1kdidiT⊗∑j(i)sjT     (3)
 MathType@MTEF@5@5@+=feaafiart1ev1aaatCvAUfKttLearuWrP9MDH5MBPbIqV92AaeXatLxBI9gBaebbnrfifHhDYfgasaacH8akY=wiFfYdH8Gipec8Eeeu0xXdbba9frFj0=OqFfea0dXdd9vqai=hGuQ8kuc9pgc9s8qqaq=dirpe0xb9q8qiLsFr0=vr0=vr0dc8meaabaqaciaacaGaaeqabaqabeGadaaakeaacqqGnbqtcqGH9aqpdaaeWbqaaiabbsgaKnaaBaaaleaacqqGPbqAaeqaaOGaeeizaq2aa0baaSqaaiabbMgaPbqaaiabbsfaubaaaeaacqqGPbqAcqGH9aqpcqaIXaqmaeaacqqGRbWAa0GaeyyeIuoakiabgEPiepaaqafabaGaee4Cam3aa0baaSqaaiabbQgaQbqaaiabbsfaubaaaeaacqqGQbGAcqGGOaakcqqGPbqAcqGGPaqkaeqaniabggHiLdGccaWLjaGaaCzcamaabmaabaGaeG4mamdacaGLOaGaayzkaaaaaa@4D18@

By feeding the context-sensitive autoassociative module M with signs or symptoms, the system retrieves the set of possible diseases associated with such set of symptoms, or a single diagnosis if the criteria suffice.

At resting conditions the system is grounded in an indifferent state g. If each disease was instructed only one time, in the mathematics of the model this implies the priming of the memory with a linear combination in which every disease has an equal weight

M(g⊗In×n)=∑i<di,g>di(∑j(i)sj)T=∑idi(∑j(i)sj)T     (4)
 MathType@MTEF@5@5@+=feaafiart1ev1aaatCvAUfKttLearuWrP9MDH5MBPbIqV92AaeXatLxBI9gBaebbnrfifHhDYfgasaacH8akY=wiFfYdH8Gipec8Eeeu0xXdbba9frFj0=OqFfea0dXdd9vqai=hGuQ8kuc9pgc9s8qqaq=dirpe0xb9q8qiLsFr0=vr0=vr0dc8meaabaqaciaacaGaaeqabaqabeGadaaakeaacqqGnbqtcqGGOaakcqqGNbWzcqGHxkcXcqqGjbqsdaWgaaWcbaGaeeOBa4Maey41aqRaeeOBa4gabeaakiabcMcaPiabg2da9maaqafabaGaeyipaWJaeeizaq2aaSbaaSqaaiabbMgaPbqabaGccqGGSaalcqqGNbWzcqGH+aGpcqqGKbazdaWgaaWcbaGaeeyAaKgabeaaaeaacqqGPbqAaeqaniabggHiLdGccqGGOaakdaaeqbqaaiabbohaZnaaBaaaleaacqqGQbGAaeqaaaqaaiabbQgaQjabcIcaOiabbMgaPjabcMcaPaqab0GaeyyeIuoakiabcMcaPmaaCaaaleqabaGaeeivaqfaaOGaeyypa0ZaaabuaeaacqqGKbazdaWgaaWcbaGaeeyAaKgabeaaaeaacqqGPbqAaeqaniabggHiLdGccqGGOaakdaaeqbqaaiabbohaZnaaBaaaleaacqqGQbGAaeqaaaqaaiabbQgaQjabcIcaOiabbMgaPjabcMcaPaqab0GaeyyeIuoakiabcMcaPmaaCaaaleqabaGaeeivaqfaaOGaaCzcaiaaxMaadaqadaqaaiabisda0aGaayjkaiaawMcaaaaa@6BBD@

where g=∑idi
 MathType@MTEF@5@5@+=feaafiart1ev1aaatCvAUfKttLearuWrP9MDH5MBPbIqV92AaeXatLxBI9gBaebbnrfifHhDYfgasaacH8akY=wiFfYdH8Gipec8Eeeu0xXdbba9frFj0=OqFfea0dXdd9vqai=hGuQ8kuc9pgc9s8qqaq=dirpe0xb9q8qiLsFr0=vr0=vr0dc8meaabaqaciaacaGaaeqabaqabeGadaaakeaacqqGNbWzcqGH9aqpdaaeqbqaaiabbsgaKnaaBaaaleaacqqGPbqAaeqaaaqaaiabbMgaPbqab0GaeyyeIuoaaaa@354C@ and I is the nxn identity matrix. From (4) it is evident that, after the priming, the context-dependent memory becomes a classical memory associating symptoms with diseases. If a set of sufficient concurrent signs and symptoms is presented to the waiting memory (σ = ∑s), after iteration, a final diagnosis results.

**Figure 1:**
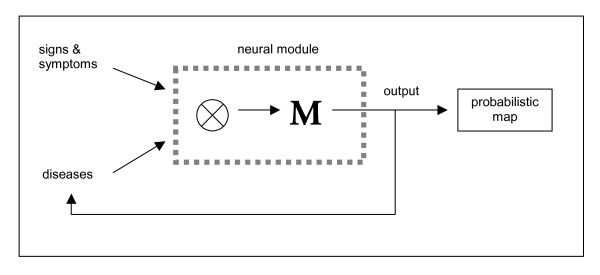
**A module with diagnostic abilities**. The neural module receives the input of two vectors: one representing the set of possible diseases up to the moment and the other vector corresponding to a new sign, symptom or laboratory result. The action of the neurons that constitute the neural module can be divided into two sequential steps: the Kronecker product of these two entries and the association of this stimulus with an output activity pattern. This output vector is a linear combination of a narrower set of disease vectors that can be reinjected if a new clinical data arrives or can be processed to obtain the probability attributable to each diagnostic decision.

It is important to point out that if the sets {s_j(i)_} corresponding to each disease were disjoint sets, then any single symptom s _j(i) _would be patognomonical and sufficient to univocally diagnose d_i_. Otherwise, the output will be a linear combination of possible diseases, each one weighed according to the scalar product between the set of actual symptoms (σ) and the set of symptoms corresponding to each different disease: ∑i<∑j(i)sj
 MathType@MTEF@5@5@+=feaafiart1ev1aaatCvAUfKttLearuWrP9MDH5MBPbIqV92AaeXatLxBI9gBaebbnrfifHhDYfgasaacH8akY=wiFfYdH8Gipec8Eeeu0xXdbba9frFj0=OqFfea0dXdd9vqai=hGuQ8kuc9pgc9s8qqaq=dirpe0xb9q8qiLsFr0=vr0=vr0dc8meaabaqaciaacaGaaeqabaqabeGadaaakeaadaaeqbqaaiabgYda8maaqafabaGaee4Cam3aaSbaaSqaaiabbQgaQbqabaaabaGaeeOAaOMaeiikaGIaeeyAaKMaeiykaKcabeqdcqGHris5aaWcbaGaeeyAaKgabeqdcqGHris5aaaa@3A9E@, σ > d_i_. See Figure 1 and its legend. Forcing the sum of scalar products to unity, this output provides a probabilistic mapping of the possible diseases associated with the clinical presentation.

### NUMERICAL EXAMPLE

#### How to instruct the memory

**Figure 2:**
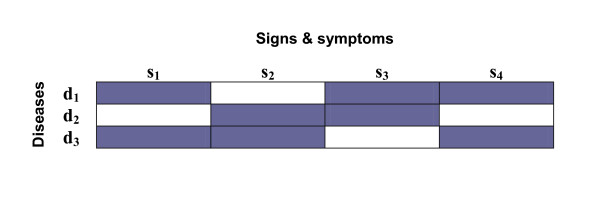
**Three diseases with their corresponding signs**. The set of signs and symptoms associated with three different diseases instructed in the memory of the numerical example according to equation (1).

Let us illustrate how to instruct the memory with a minimal numerical example. Consider the set of three diseases and its characteristic symptoms shown in Figure 2. The first task is to codify the sets of signs and diseases with orthogonal vectors, for which we will use the following orthogonal matrices.

Diseases         Signs&symptoms[100010001]0.5*[11111−11−111−1−11−1−11]d1d2d3         s1s2s3s4
 MathType@MTEF@5@5@+=feaafiart1ev1aaatCvAUfKttLearuWrP9MDH5MBPbIqV92AaeXatLxBI9gBamXvP5wqSXMqHnxAJn0BKvguHDwzZbqegyvzYrwyUfgarqqtubsr4rNCHbGeaGqiA8vkIkVAFgIELiFeLkFeLk=iY=Hhbbf9v8qqaqFr0xc9pk0xbba9q8WqFfeaY=biLkVcLq=JHqVepeea0=as0db9vqpepesP0xe9Fve9Fve9GapdbaqaaeGacaGaaiaabeqaamqadiabaaGcbaqbaeqabmGaaaqaaGWabiaa=reacaWFPbGaa83Caiaa=vgacaWFHbGaa83Caiaa=vgacaWFZbaaeaqabeaacaWFGaGaa8hiaiaa=bcacaWFGaGaa8hiaiaa=bcacaWFGaGaiaiG4eaaa83uaiacaciobaaa=LgacGaGaItaaaWFNbGaiaiG4eaaa8NBaiacaciobaaa=nhacGaGaItaaaWFMaaabaGaa8hiaiaa=bcacaWFGaGaa8hiaiaa=bcacaWFGaGaa8hiaiaa=bcacaWFZbGaa8xEaiaa=1gacaWFWbGaa8hDaiaa=9gacaWFTbGaa83CaaaabaWaamWaaeaafaqabeWadaafbaGaeGymaedabaGaeGimaadabaGaeGimaadabaGaeGimaadabaGaeGymaedabaGaeGimaadabaGaeGimaadabaGaeGimaadabaGaeGymaedaaaGaay5waiaaw2faaaqaaiabicdaWiabc6caUiabiwda1iabcQcaQmaadmaabaqbaeqabqabaaavaeaacqaIXaqmaeaacqaIXaqmaeaacqaIXaqmaeaacqaIXaqmaeaacqaIXaqmaeaacqGHsislcqaIXaqmaeaacqaIXaqmaeaacqGHsislcqaIXaqmaeaacqaIXaqmaeaacqaIXaqmaeaacqGHsislcqaIXaqmaeaacqGHsislcqaIXaqmaeaacqaIXaqmaeaacqGHsislcqaIXaqmaeaacqGHsislcqaIXaqmaeaacqaIXaqmaaaacaGLBbGaayzxaaaabaqbaeqabeWaaaqaaiaa=rgadaWgaaWcbaGaa8xmaaqabaaakeaacaWFKbWaaSbaaSqaaiaa=jdaaeqaaaGcbaGaa8hzamaaBaaaleaacaWFZaaabeaaaaaakeaafaqabeqaeaaaaeaacGaGasMaaaWFZbWaiaiGKjaaaSbaaSqaiaiGKjaaaiacacizcaaa=fdaaeqcacizcaaaaaGcbaGaiaiGWgaaa83CamacaciSbaaaBaaaleacaciSbaaacGaGacBaaaWFYaaabKaGacBaaaaaaOqaaiacaciJbaaa=nhadGaGaYyaaaWgaaWcbGaGaYyaaaGaiaiGmgaaa83maaqajaiGmgaaaaaakeGacaG7aaGzbiaa=bcacGaGaYvaaaWFZbWaiaiGCfaaaSbaaSqaiaiGCfaaaiacacixbaaa=rdaaeqcacixbaaaaaaaaaaaaa@B4B3@

According to the table and equation (1), we instruct the memory by adding a matrix for each disease. For the first disease we have d_1_d_1_^T ^⊗ (s_1 _+ s_3 _+ s_4_)^T^

[100000000]⊗[1.50.5−0.50.5]=     =[1.50.5−0.50.500000000000000000000000000000000]
 MathType@MTEF@5@5@+=feaafiart1ev1aaatCvAUfKttLearuWrP9MDH5MBPbIqV92AaeXatLxBI9gBaebbnrfifHhDYfgasaacH8akY=wiFfYdH8Gipec8Eeeu0xXdbba9frFj0=OqFfea0dXdd9vqai=hGuQ8kuc9pgc9s8qqaq=dirpe0xb9q8qiLsFr0=vr0=vr0dc8meaabaqaciaacaGaaeqabaqabeGadaaakeaafaqaaeGabaaabaWaamWaaeaafaqabeWadaaabaGaeGymaedabaGaeGimaadabaGaeGimaadabaGaeGimaadabaGaeGimaadabaGaeGimaadabaGaeGimaadabaGaeGimaadabaGaeGimaadaaaGaay5waiaaw2faaiabgEPielabcUfaBvaabeqabqaaaaqaaiabigdaXiabc6caUiabiwda1aqaaiabicdaWiabc6caUiabiwda1aqaaiabgkHiTiabicdaWiabc6caUiabiwda1aqaaiabicdaWiabc6caUiabiwda1aaacqGGDbqxcqGH9aqpaeaacaWLjaGaaCzcaiabg2da9maadmaabaqbaeqabmadaaaaaaqaaiabigdaXiabc6caUiabiwda1aqaaiabicdaWiabc6caUiabiwda1aqaaiabgkHiTiabicdaWiabc6caUiabiwda1aqaaiabicdaWiabc6caUiabiwda1aqaaiabicdaWaqaaiabicdaWaqaaiabicdaWaqaaiabicdaWaqaaiabicdaWaqaaiabicdaWaqaaiabicdaWaqaaiabicdaWaqaaiabicdaWaqaaiabicdaWaqaaiabicdaWaqaaiabicdaWaqaaiabicdaWaqaaiabicdaWaqaaiabicdaWaqaaiabicdaWaqaaiabicdaWaqaaiabicdaWaqaaiabicdaWaqaaiabicdaWaqaaiabicdaWaqaaiabicdaWaqaaiabicdaWaqaaiabicdaWaqaaiabicdaWaqaaiabicdaWaqaaiabicdaWaqaaiabicdaWaqaaiabicdaWaqaaiabicdaWaqaaiabicdaWaqaaiabicdaWaaaaiaawUfacaGLDbaaaaaaaa@7726@

In the same way we will have two other matrices for the other diseases. The sum of the three matrices constitutes the memory M.

M=[1.50.5−0.50.5000000000000100−10000000000001.5−0.50.50.5]
 MathType@MTEF@5@5@+=feaafiart1ev1aaatCvAUfKttLearuWrP9MDH5MBPbIqV92AaeXatLxBI9gBaebbnrfifHhDYfgasaacH8akY=wiFfYdH8Gipec8Eeeu0xXdbba9frFj0=OqFfea0dXdd9vqai=hGuQ8kuc9pgc9s8qqaq=dirpe0xb9q8qiLsFr0=vr0=vr0dc8meaabaqaciaacaGaaeqabaqabeGadaaakeaacqqGnbqtcqGH9aqpdaWadaqaauaabeqadWaaaaaaaeaacqaIXaqmcqGGUaGlcqaI1aqnaeaacqaIWaamcqGGUaGlcqaI1aqnaeaacqGHsislcqaIWaamcqGGUaGlcqaI1aqnaeaacqaIWaamcqGGUaGlcqaI1aqnaeaacqaIWaamaeaacqaIWaamaeaacqaIWaamaeaacqaIWaamaeaacqaIWaamaeaacqaIWaamaeaacqaIWaamaeaacqaIWaamaeaacqaIWaamaeaacqaIWaamaeaacqaIWaamaeaacqaIWaamaeaacqaIXaqmaeaacqaIWaamaeaacqaIWaamaeaacqGHsislcqaIXaqmaeaacqaIWaamaeaacqaIWaamaeaacqaIWaamaeaacqaIWaamaeaacqaIWaamaeaacqaIWaamaeaacqaIWaamaeaacqaIWaamaeaacqaIWaamaeaacqaIWaamaeaacqaIWaamaeaacqaIWaamaeaacqaIXaqmcqGGUaGlcqaI1aqnaeaacqGHsislcqaIWaamcqGGUaGlcqaI1aqnaeaacqaIWaamcqGGUaGlcqaI1aqnaeaacqaIWaamcqGGUaGlcqaI1aqnaaaacaGLBbGaayzxaaaaaa@6427@

#### How the system works

See also Figure 1 and its legend.

Time step 1

Initial state of the system: Indifferent vector g^T ^= (d_1 _+ d_2 _+ d_3_) = [1 1 1]

A first clinical data (s_3_) arrives: s_3 _^T ^= [0.5 0.5 -0.5 -0.5]

Preprocessing of input vectors is performed: h = g ⊗ s_3_

h^T ^= [0.5 0.5 -0.5 -0.5 0.5 0.5 -0.5 -0.5 0.5 0.5 -0.5 -0.5]

Resulting associated output: Mh (a linear combination of possible diagnoses)

output(1)=[110]
 MathType@MTEF@5@5@+=feaafiart1ev1aaatCvAUfKttLearuWrP9MDH5MBPbIqV92AaeXatLxBI9gBaebbnrfifHhDYfgasaacH8akY=wiFfYdH8Gipec8Eeeu0xXdbba9frFj0=OqFfea0dXdd9vqai=hGuQ8kuc9pgc9s8qqaq=dirpe0xb9q8qiLsFr0=vr0=vr0dc8meaabaqaciaacaGaaeqabaqabeGadaaakeaacqqGVbWBcqqG1bqDcqqG0baDcqqGWbaCcqqG1bqDcqqG0baDcqGGOaakcqaIXaqmcqGGPaqkcqGH9aqpdaWadaqaauaabeqadeaaaeaacqaIXaqmaeaacqaIXaqmaeaacqaIWaamaaaacaGLBbGaayzxaaaaaa@3DAF@

Resulting probabilistic map (each coefficient of the output vector is divided by the sum of them all):

prob(1)=[0.50.50]
 MathType@MTEF@5@5@+=feaafiart1ev1aaatCvAUfKttLearuWrP9MDH5MBPbIqV92AaeXatLxBI9gBaebbnrfifHhDYfgasaacH8akY=wiFfYdH8Gipec8Eeeu0xXdbba9frFj0=OqFfea0dXdd9vqai=hGuQ8kuc9pgc9s8qqaq=dirpe0xb9q8qiLsFr0=vr0=vr0dc8meaabaqaciaacaGaaeqabaqabeGadaaakeaacqqGWbaCcqqGYbGCcqqGVbWBcqqGIbGycqGGOaakcqaIXaqmcqGGPaqkcqGH9aqpdaWadaqaauaabeqadeaaaeaacqaIWaamcqGGUaGlcqaI1aqnaeaacqaIWaamcqGGUaGlcqaI1aqnaeaacqaIWaamaaaacaGLBbGaayzxaaaaaa@3E59@

Time step 2

A new symptom (s_2_) arrives: s_2 _^T ^= [0.5 -0.5 0.5 -0.5]

Preprocessing of input vectors is performed: h = output(1) ⊗ s_2_

h^T ^= [0.5 -0.5 0.5 -0.5 0.5 -0.5 0.5 -0.5 0 0 0 0]

Resulting associated output (Mh):

output(2)=[010]
 MathType@MTEF@5@5@+=feaafiart1ev1aaatCvAUfKttLearuWrP9MDH5MBPbIqV92AaeXatLxBI9gBaebbnrfifHhDYfgasaacH8akY=wiFfYdH8Gipec8Eeeu0xXdbba9frFj0=OqFfea0dXdd9vqai=hGuQ8kuc9pgc9s8qqaq=dirpe0xb9q8qiLsFr0=vr0=vr0dc8meaabaqaciaacaGaaeqabaqabeGadaaakeaacqqGVbWBcqqG1bqDcqqG0baDcqqGWbaCcqqG1bqDcqqG0baDcqGGOaakcqaIYaGmcqGGPaqkcqGH9aqpdaWadaqaauaabeqadeaaaeaacqaIWaamaeaacqaIXaqmaeaacqaIWaamaaaacaGLBbGaayzxaaaaaa@3DAF@

Resulting probabilistic map:

prob(1)=[010]
 MathType@MTEF@5@5@+=feaafiart1ev1aaatCvAUfKttLearuWrP9MDH5MBPbIqV92AaeXatLxBI9gBaebbnrfifHhDYfgasaacH8akY=wiFfYdH8Gipec8Eeeu0xXdbba9frFj0=OqFfea0dXdd9vqai=hGuQ8kuc9pgc9s8qqaq=dirpe0xb9q8qiLsFr0=vr0=vr0dc8meaabaqaciaacaGaaeqabaqabeGadaaakeaacqqGWbaCcqqGYbGCcqqGVbWBcqqGIbGycqGGOaakcqaIXaqmcqGGPaqkcqGH9aqpdaWadaqaauaabeqadeaaaeaacqaIWaamaeaacqaIXaqmaeaacqaIWaamaaaacaGLBbGaayzxaaaaaa@3AA3@

#### Final result

The system has arrived to an only final diagnosis that corresponds to disease 2.

### REAL DATA APPLICATION – diagnosing late-onset neonatal sepsis

Late-onset sepsis (invasive infection occurring in neonates after 3 days of age) is an important and severe problem in infants hospitalized in neonatal intensive care units (NICUs) [22]. The clinical signs of infection in the newborn are variable, and the earliest manifestations are often subtle and nonspecific. In the presence of a clinical suspicion of sepsis an early and accurate diagnosis algorithm would be of outstanding value but is not yet available [23]. In a recent retrospective study that included 47 neonates with clinical diagnosis of suspected sepsis, Martell and collaborators [24] assessed a group of clinical and laboratory variables – surgical history, metabolic acidosis, hepatomegalia, abnormal white blood cell (WBC) count, hyperglycemia and thrombocytopenia-determining their sensitivity, specificity, likelihood ratio and post-test probability. Sepsis was defined as a positive result on one or more blood cultures in a neonate with clinical diagnosis of suspected sepsis. A prevalence of 34% was found for their NICU.

We instructed a context-dependent autoassociative memory according to equation (3) with data published in [24] in order to evaluate its capacity to recognize patients with or without sepsis. As a test-set, we used 15 cases of suspected neonatal sepsis coming from the same NICU (personal observations of one of us-AP). From equation (3) it is clear that the different clinical presentation of the individual cases are added up and resumed in the vector (∑j(i)sjT
 MathType@MTEF@5@5@+=feaafiart1ev1aaatCvAUfKttLearuWrP9MDH5MBPbIqV92AaeXatLxBI9gBaebbnrfifHhDYfgasaacH8akY=wiFfYdH8Gipec8Eeeu0xXdbba9frFj0=OqFfea0dXdd9vqai=hGuQ8kuc9pgc9s8qqaq=dirpe0xb9q8qiLsFr0=vr0=vr0dc8meaabaqaciaacaGaaeqabaqabeGadaaakeaadaaeqbqaaiabbohaZnaaDaaaleaacqqGQbGAaeaacqqGubavaaaabaGaeeOAaOMaeiikaGIaeeyAaKMaeiykaKcabeqdcqGHris5aaaa@374E@) representing the characteristic signs of each illness condition. We trained the memory instructing two terms d_i _corresponding to the two final diagnoses of confirmed sepsis and absence of sepsis.

M = [septic] [septic]^T ^⊗ [attributes _ septic]^T ^+ [healthy] [healthy]^T ^⊗ [attributes _ healthy]^T^

The column vectors used for the septic and healthy conditions were [1 0]^T ^and [0 1]^T ^respectively.

**Figure 3:**
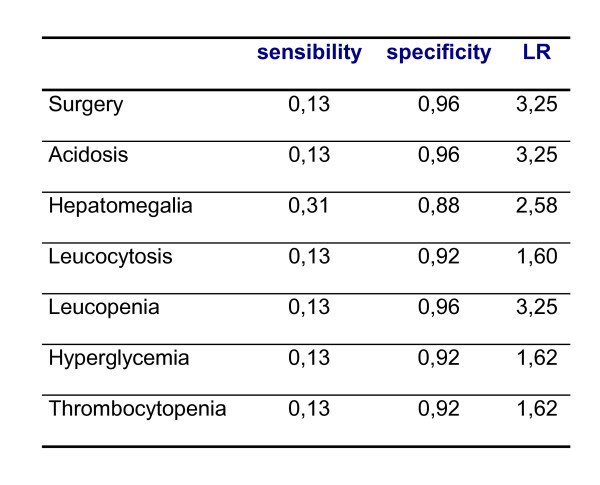
**Data from the study of Martell and collaborators [24]**. The total number of neonates with suspected late-onset sepsis was N = 47 (septic: E = 16; non-septic: NE = 31). Metabolic acidosis was defined for patients with adequate ventilation; WBC count was considered normal within 5.000–25.000; thrombocytopenia was defined for platelet counts <40.000.

The column vectors with the attributes corresponding to the septic and non-septic patients were generated from the available data as follows. For each one of the variables studied in [24] (see Figure 3) we reconstructed the values of the true positive (TP), false positive (FP), true negative (TN) and false negative (FN) number of patients:

TP = sensitivity × E

FP = (sensitivity/LR) × NE

TN = specificity × NE

FN = N - (TP+FP+TN).

**Figure 4:**
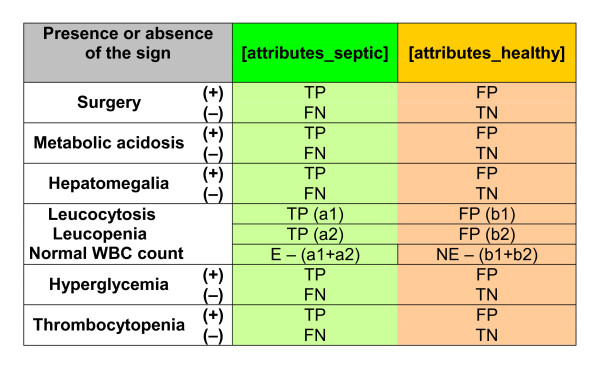
**Attributes' vectors for the septic and non-septic groups**. For each variable, true positive (TP), false positive (FP), true negative (TN) and false negative (FN) were calculated: TP = sensitivity × E; FP = (sensitivity/LR) × NE; TN = specificity × NE; FN = N - (TP+FP+TN).

These values became the coefficients of the two thirteen-dimensional column vectors [attributes_septic] and [attributes_healthy]. This procedure is shown in Figure 4. Finally, after normalization, the vectors used for the instruction of the memory M are

[attributes_septic]^T ^= [0.0604 0.4225 0.0604 0.4225 0.1509 0.3320 0.0604 0.0604 0.3621 0.0604 0.4225 0.0604 0.4225]

[attributes_healthy]^T ^= [0.0142 0.4248 0.0142 0.4248 0.0566 0.3823 0.0354 0.0177 0.3859 0.0283 0.4106 0.0283 0.4106].

The memory M resumes the cumulated experience in suspected late-onset sepsis of this particular NICU through the clinical presentations of one year hospitalized neonates.

**Figure 5:**
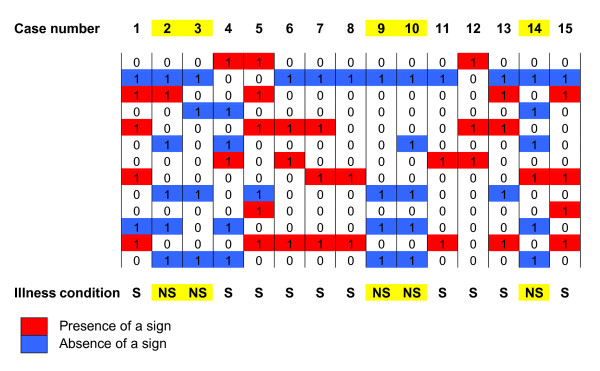
**Test set**. For each one of the 15 neonates of the test set, the thirteen-dimensional column vector has ones in the coefficients corresponding to the position of the confirmed presence or absence of the signs already shown in Figure 4. The final diagnosis is shown at the bottom of the column (S: sepsis; NS: no sepsis).

To test our system we presented to the memory a set of 15 personal clinical observations of neonates with the clinical diagnosis of suspected sepsis hospitalized in the same NICU. We coded the thirteen attributes with the canonical basis vectors (the columns of a 13-dimensional identity matrix). For example, the presence of metabolic acidosis was represented with [0 01 0 0 0 0 0 0 0 0 0 0]^T ^and the absence of acidosis with the vector [0 0 01 0 0 0 0 0 0 0 0 0]^T^. For each patient of the test set we added the vectors corresponding to the confirmed presence or absence of any sign. These 15 vectors representing the clinical presentation of the neonates with the diagnosis of suspected sepsis are shown in Figure 5.

The classification of each patient was obtained as follows:

i) The vector with the clinical presentation is presented to the memory M. The output, [result_vector], is a linear combination of the vectors septic [1 0]^T ^and healthy [0 1]^T^:

[result_vector] = M * ([indifferent_vector] ⊗ [clinical presentation])

The [indifferent_vector] is the sum of septic and healthy vectors: [1 1]^T^.

ii) A diagnosis results from the evaluation of the coefficients of the two-dimensional [result_vector]. If the first coefficient is greater than the second the case is classified as sepsis. If the second coefficient is the largest the patient is classified as non-septic.

## Results

### A context-dependent memory model acting as a minimal expert system

In this work we show a minimal, context-dependent, memory nucleus able to support diagnostic abilities. Our expert system consists of an autoassociative memory with overlapping contexts and feedback loop that makes the output able to be reinjected into the memory at the next time step (Figure 1).

A memory M acting as a basic expert system is a matrix (equation 3)

M=∑i=1kdidiT⊗∑j(i)sjT,
 MathType@MTEF@5@5@+=feaafiart1ev1aaatCvAUfKttLearuWrP9MDH5MBPbIqV92AaeXatLxBI9gBaebbnrfifHhDYfgasaacH8akY=wiFfYdH8Gipec8Eeeu0xXdbba9frFj0=OqFfea0dXdd9vqai=hGuQ8kuc9pgc9s8qqaq=dirpe0xb9q8qiLsFr0=vr0=vr0dc8meaabaqaciaacaGaaeqabaqabeGadaaakeaacqqGnbqtcqGH9aqpdaaeWbqaaiabbsgaKnaaBaaaleaacqqGPbqAaeqaaOGaeeizaq2aa0baaSqaaiabbMgaPbqaaiabbsfaubaaaeaacqqGPbqAcqGH9aqpcqaIXaqmaeaacqqGRbWAa0GaeyyeIuoakiabgEPiepaaqafabaGaee4Cam3aa0baaSqaaiabbQgaQbqaaiabbsfaubaaaeaacqqGQbGAcqGGOaakcqqGPbqAcqGGPaqkaeqaniabggHiLdGccqGGSaalaaa@4A37@

where the d_i _are column vectors mapping k different diseases (the set {d} is chosen to be orthonormal), s_j(i) _are column vectors mapping signs and symptoms accompanying the i disease (also an orthonormal set), and ⊗ is the Kronecker product [25]-see **Methods**-. Note that if d are n-dimensional vectors (n ≥ k), and s are m-dimensional, then d_i_d_i_^T^ are square symmetric matrices, and the memory M is a rectangular matrix of dimensions nxnm.

### The instruction of the expert

The cognitive functioning shown by this kind of neural network model is based on the establishment of context-dependent associations. The instruction of the expert therefore consists in the instruction of the memory that stores these associations.

Each disease is instructed to the memory together with its characteristic signs and symptoms (these can include the results of laboratory exams, imaging studies, etc). For this to be done, the first step is to code each disease to be instructed with a different orthonormal vector. The same must be done with the set of signs, symptoms and paraclinical results that could accompany that set of diseases, also coding them with different column vectors of any orthonormal basis of adequate dimension.

Once the signs and symptoms corresponding to each disease have been identified and expressed as orthogonal vectors, the construction of the memory can commence. According to equation (1) this instruction consists in the superposition (the addition) of different rectangular matrices, each one corresponding to a different disease.

The instruction of the memory can be developed along two different paths. a. *Learning from the textbook*. In this case, the expert is instructed according to the updated academic knowledge of each disease. One first disease is taken, which is coded by the column vector d_i_, and the outer product of this vector is made by itself (a square matrix is constructed that contains this autoassociation). At the same time, all the signs and symptoms characteristic of this disease are identified and the vectors coding them are added up (∑j(i)sj
 MathType@MTEF@5@5@+=feaafiart1ev1aaatCvAUfKttLearuWrP9MDH5MBPbIqV92AaeXatLxBI9gBaebbnrfifHhDYfgasaacH8akY=wiFfYdH8Gipec8Eeeu0xXdbba9frFj0=OqFfea0dXdd9vqai=hGuQ8kuc9pgc9s8qqaq=dirpe0xb9q8qiLsFr0=vr0=vr0dc8meaabaqaciaacaGaaeqabaqabeGadaaakeaadaaeqbqaaiabbohaZnaaBaaaleaacqqGQbGAaeqaaaqaaiabbQgaQjabcIcaOiabbMgaPjabcMcaPaqab0GaeyyeIuoaaaa@361E@). Finally the Kronecker product between the square matrix and the transpose of the vector-sum is performed. An analogous procedure is accomplished for any pathology. Each new resulting rectangular matrix of dimension nxnm is added to the previous ones already stored in memory M (a minimal numerical example is presented in section **Methods**-How to instruct the memory-). b. *Learning by experience*. This is a case-based way of instructing the memory. It allows the expert to progressively capture the prevalence of the different diseases in a community. Once finalized the previous instruction, the memory is fed with the actual clinical findings of each particular patient assisted by the physician, attributing this particular constellation of signs and symptoms to the corresponding final diagnosis. The matrices resulting from new patients are progressively added to the memory. This type of representation implies two essential distinctions from the previous *learning-from-the-textbook *memory. Pathologies are not equally weighed in the memory but their representations depend on the frequency of presentation of cases in the population. In addition, for each disease the different symptoms also are not equally weighed: those corresponding to the more frequent clinical presentations will be strengthened.

### Medical queries

Once the training phase is finalized, the system is ready to be used. The presentation of a first sign or symptom initiates a medical query. The availability of a new clinical or laboratory finding causes the expert to advance one more step in its diagnostic decision. Although we have many new signs and symptoms, in order to obtain a progressive narrowing of the set of possible diagnoses they must be presented to the expert one per time. At each step, the new data are entered into the memory along with the set of possible diagnoses until that moment. Finally, if the whole set of signs and symptoms available until the moment is sufficient, the system will arrive to a unique diagnosis.

We then follow the system operation. The starting point is when the first clinical data appears. The vector corresponding to this symptom is multiplied by means of the Kronecker product times the vector that represents the set of possible diagnoses (in the starting point it is an indifferent vector). If the memory was instructed with equally-weighed pathologies the indifferent vector is the sum of all the vectors of diseases stored in the memory. If, on the contrary, the memory was instructed on the basis of individual cases, the indifferent vector will be the same linear combination of the vector diseases stored (the weight of each disease corresponds to the one of its frequency of presentation). The resulting column vector is now multiplied by the memory matrix. The exit vector contains either a univocal diagnosis (if the clinical data are sufficient) or a certain linear combination of vectors corresponding to several diseases. If a unique diagnosis was not arrived at, when one has a new sign or symptom, its corresponding vector will enter the memory after making its Kronecker product by the exit vector of the previous step. The process is repeated and stops when a final diagnosis is reached or when new clinical data is not available (see the continuation of the numerical example in section **Methods**-How the system works-).

Even if at a certain state a final diagnosis has not been reached, the outcome of the system nevertheless represents a probabilistic mapping of the possible diagnoses, each one with its respective probability in agreement with the data available until the moment. In order to obtain such a map in a direct way it is convenient to choose as disease vectors the columns of an identity matrix of suitable dimension. In that case, in each exit vector the positions of the coefficients different from zero mark the different possible diagnoses. Applying a normalization to this exit vector in such a way that the sum of their components is one, the value of each coefficient different from zero represents the probability of each one of those diagnoses. Otherwise, these probabilities can be obtained by multiplying the exit vector by the orthonormal matrix that codifies the diseases.

### A reduced model for the diagnosis of late-onset neonatal sepsis

The system described in section **Methods **classified the patients of the test-set (N = 15) as follows (S = sepsis; NS = non-septic):

123456789101112131415SNSNSSSSSSNSNSSSSSS
 MathType@MTEF@5@5@+=feaafiart1ev1aaatCvAUfKttLearuWrP9MDH5MBPbIqV92AaeXatLxBI9gBaebbnrfifHhDYfgasaacH8akY=wiFfYdH8Gipec8Eeeu0xXdbba9frFj0=OqFfea0dXdd9vqai=hGuQ8kuc9pgc9s8qqaq=dirpe0xb9q8qiLsFr0=vr0=vr0dc8meaabaqaaiaacaGaaeqabaqabeGadaaakeaafaqabeGapqaaaaaabaGaeGymaedabaGaeGOmaidabaGaeG4mamdabaGaeGinaqdabaGaeGynaudabaGaeGOnaydabaGaeG4naCdabaGaeGioaGdabaGaeGyoaKdabaGaeGymaeJaeGimaadabaGaeGymaeJaeGymaedabaGaeGymaeJaeGOmaidabaGaeGymaeJaeG4mamdabaGaeGymaeJaeGinaqdabaGaeGymaeJaeGynaudabaGaem4uamfabaGaemOta4Kaem4uamfabaGaemOta4Kaem4uamfabaGaem4uamfabaGaem4uamfabaGaem4uamfabaGaem4uamfabaGaem4uamfabaGaemOta4Kaem4uamfabaGaemOta4Kaem4uamfabaGaem4uamfabaGaem4uamfabaGaem4uamfabaGaem4uamfabaGaem4uamfaaaaa@5744@

**Figure 6:**
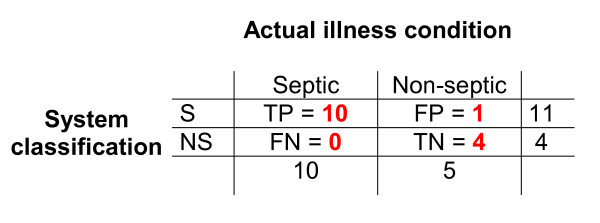
**Associative memory classification**. The 15 cases tested (actual sepsis: 10; non-septic: 5) were classified as positive (S) or negative (NS) by the neural network. TP = true positive; FP = false positive; TN = true negative and FN = false negative. A total of 11 neonates were tested positive, and 4 negative. The sensitivity was 100% and the specificity 80%.

Comparing this classification with the actual illness condition of the patients-shown in Figure 5 – it results that only patient 14 was misdiagnosed. The 2 × 2 table shown in Figure 6 resumes the behaviour of our diagnostic system. The sensitivity was 100% and the specificity 80%. The likelihood ratio (LR = TP/FP) was 5. Using this set of variables as input data, the performance of the system in the classification task can be evaluated as very good. It reached a high accuracy ((TP+TN)/N) of 93.3%, and a Cohen's kappa index of 0.84. (Kappa = (Accuracy - A_chance)/(1-A_chance), where A_chance is the accuracy expected by chance. A_chance = (<TP>+<TN>)/N) where <TP> = (TP+FP)(TP+FN)/N and <TN> = (FN+TN)(FP+TN)/N).

## Discussion and conclusions

We have shown here that context-dependent associative memories could act as medical decision support systems. The system implies the previous coding of a set of diseases and its corresponding semiologic findings in individual basis of orthogonal vectors. The model presented in this communication is only a minimal module able to evaluate the probabilities of different diagnoses when a set of signs and symptoms is presented to it.

This expert system based on an associative memory shares with programs using artificial intelligence a great capacity to quickly narrow the number of diagnostic possibilities [1]. Also, it is able to cope with variations in the way that a disease can present itself.

**Figure 7:**
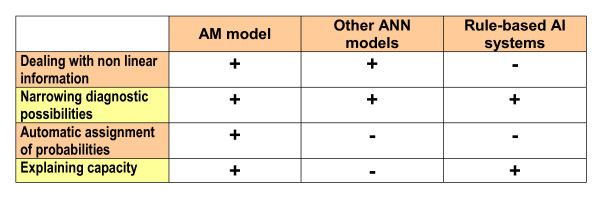
**Outstanding characteristics of different models**. AM: associative memory; ANN: artificial neural network; AI: artificial intelligence.

A clear advantage of this system is that the probability assignment to the different diagnostic possibilities in any particular clinical situation does not have to be arbitrarily assigned by the specialist, but is automatically provided by the system, in agreement with the acquired experience. In this sense, this neural network model is akin to statistical pattern recognition [1]. However, neither programs based on simple matching strategies nor most used neural network models are able to explain to the physician how they have reached their conclusions. On the contrary, the operation of this system, that unveils the underlying associative structure of human cognition, is transparent. Obviously, it must be understood that this is not the unique mechanism involved in human decision making. The relevant properties of this associative memory model are summarized in Figure 7 in comparison to other neural network models and rule-based artificial intelligence systems.

Beginning with a textbook-instructed memory, the system evolves accommodating (superimposing in the memory) new manifestations of disease gathered over time. This process of continued network education based on empirical evidence leads to databases representative of the different patient populations with its own geo-demographical characteristics.

This model can be easily improved in various directions. The functioning of the system described up to now can be considered a passive phase (in the sense that it consists on an automatic evaluation of the available information). By adding another module to the system, consisting of a simple memory that associates diseases with the set of its findings, the expert can enhance its diagnostic performance. Remaining two or three different diagnostic hypothesis within the previous passive phase of diagnosis refinement, this new module can be fed with the vectors mapping each one of these diseases to elicit its associated set of clinical findings. The set of absent features supporting one or the other disease determines what information must be sought next.

Another important expansion of the expert allows giving up the strong assumption that all the findings correspond to a unique disease. Our context-dependent memory stops and gives a null vector when contradictory data are proportioned. To prevent such behaviour, a module akin to a novelty filter could be interposed within the recursion with the following properties: if a vector with only zero coefficients arrives, this module associates the whole set of diseases, avoiding lying aside relevant diagnoses and concurrent pathologies. However, this theme needs further investigation: as for almost every expert system [26], the clustering of findings and their attribution either to only one disease or to several disorders is a major challenge.

The primary implementation of a reduced version of the model with the aim of classifying septic or non-septic neonates showed the highly satisfactory capacity of the model to be applied to real data. We conclude that context-sensitive associative memory model is a promising alternative in the development of accuracy diagnostic tools. We expect that its easy implementation stimulate groups of medical informatics to develop this expert system at real scale.

## Competing interests

The authors declare that they have no competing interests.

## Authors' contributions

AP conceived the application of the model to medical diagnosis, drafted the manuscript and carried out the implementation with real data of neonates with suspected late-onset sepsis. FO participated in the elaboration of the numerical examples, computational programs and the discussion of the model. Both authors read and approved the final manuscript.

## Pre-publication history

The pre-publication history for this paper can be accessed here:


